# Supplementary material for: Prevalence of pfdhfr-pfdhps Sextuple and Gametocyte-Associated Quintuple Sulfadoxine-Pyrimethamine Resistance Mutations in Plasmodium falciparum Isolates from Pregnant Women in Mozambique
Source: Pathogens. 2026 May 7;15(5):504. doi: 10.3390/pathogens15050504 (PMC13209286; doi:10.3390/pathogens15050504)
Supplement: Supplementary file 1 [file pathogens-15-00504-s001.zip › pathogens-4244918-supplementary.pdf]

Prevalence of *pfdhfr-pfdhps* sextuple and gametocyte-associated quintuple sulfadoxine-pyrimethamine resistance mutations in *Plasmodium falciparum* isolates from pregnant women in Mozambique

Supplementary materials

METHODS

Pfdhfr and pfdhps Genotyping - Restriction fragment length polymorphism

We analyzed single nucleotide polymorphisms at the *Pfdhfr* (N51I, C59R, S108N and I164L) and *Pfdhps* (S436F, A437G, K540E and A581G) loci by PCR-restriction fragment length polymorphism (PCR-RFLP) using primers and nested-PCR protocols described elsewhere [1,2], with minor modifications. PCR reactions were run on a Biometra T professional gradient Thermocycler (Thistle Scientific Ltd., Glasgow, UK). For the *dhfr* loci, primary PCR was performed in a 25 µL mixture containing 2 µL of DNA sample (or 5 µL for samples with a parasite density below 1 parasite/µL), 1.5 mM MgCl2, 10x PCR Buffer, 200 µM dNTP, 0.2 µL of HotStarTaq DNA polymerase and 0.2 µM of each primer. A 720-base pair (bp) sequence was amplified with primer pairs AMP1/AMP2 (Table S1) with the following settings: initial denaturation 94°C for 3 min, 45 cycles of 94°C for 30 s, 45°C for 45 s and 72°C for 45 s with a final extension of 72°C for 5 min. For the nested PCR, 2 µL of primary PCR product was added to each of two PCR mixtures to create a final volume of 50 µL containing 1.5 mM MgCl2, 10x PCR Buffer, 200 µM dNTP, 0.2 µL of HotStarTaq DNA polymerase, and 0.25 µM of each primer. The primer pairs F-M4 and M3-F/ were used to amplify codon C59R and codons N51I, S108N and I164L, respectively with the following settings: initial denaturation 94°C for 3 min; 5 cycles of 94°C for 1 min, 45°C for 2 min, and 72°C for 1 min; 35 cycles of 94°C for 1 min, 45°C for 1 min, and 72°C for 1 min; and a final extension of 72°C for 10 min.

For the *dhps* loci, a primary PCR was performed in a 50 µL mixture containing 5 µL of DNA sample, 1.5 mM MgCl2, 10x PCR Buffer, 200 µM dNTP, 0.2 µL of HotStarTaq DNA Polymerase

and 0.2 µM of each primer. A 710-bp sequence was amplified with primer pairs R2/R (Table S1) with the following settings: initial denaturation 94°C for 3 min; 45 cycles of 94°C for 1 min, 45°C for 45 s, and 72°C for 1 min; and a final extension of 72°C for 5 min. For the nested PCR, 2 µL of primary PCR product was used in a final volume of 50 µL containing PCR mixture with 1.5 mM MgCl2, 10x PCR Buffer, 200 µM dNTP, 0.2 µL of HotStarTaq DNA polymerase, and 0.2 µM of each primer pair. The following primer pairs were used: K-K/ for A437G, and K540E, J-K for S436F, and L-L/ for A581G and A613S. The settings were: initial denaturation 94°C for 2 min; 5 cycles of 94°C for 2 min, 45°C for 2 min, and 72°C for 1.5 min; 35 cycles of 94°C for 1 min, 45°C for 1 min, and 72°C for 1 min; and a final extension of 72°C for 10 min.

Five microliters (100-250 ng) of the nested PCR products containing the polymorphic regions were subjected to enzymatic digestion (including appropriate reaction buffers, *i.e.,* 10x NEBuffer A, New England Biolabs, Ipswich, MA, USA) to detect mutations at the various sites. Single enzymatic digestions were conducted in a final volume reaction of 15 µL following the manufacturer's instructions (New England Biolabs, Ipswich, MA, USA). Double enzymatic digestions at 581 codon were conducted in a final volume reaction of 50 µL. The first incubation was performed at 55°C for 5-15 min with digestion of five microliters (100-250 ng) of DNA in 10 units of BslI. Secondly, 10 units of BstUI were added into the reaction and incubated at 60°C for 5-15 min. Furthermore, double enzymatic digestions at 164 codon were conducted in a final volume reaction of 10 µL. The first incubation was performed at 37°C for 1.5 hours with digestion of five microliters (100-250 ng) of DNA in five units of DraI. Secondly, 10 units of DraI were added into the reaction and incubated at 37°C for 1.5 hours followed by enzyme inactivation at 65°C for 20 min.

Primer sequences, restriction enzyme digestion, and fragment sizes are shown in Table S1. Plasmid controls for PCR-RFLP were obtained from MR4/BEI resources "https://www.beiresources.org/MR4Home.aspx (accessed on 23 March 2026)" and were used as

wild-type and mutant controls for *pfdhfr* and *pfdhps* polymorphisms. Specifically: i) FR-3D7 (MRA199) and FR-V1/S (MRA195) used as wild-type and mutant controls for *dhfr* N51I, C59R, S108N, I164L, respectively; ii) PS-FCR (MRA192) used for *dhps* S436F and A437G wild-types; iii) PS-Dd2 (MRA193) used for *dhps* S436F mutant; iv) PS-Mali (MRA191) used for *dhps*, K540E, A581G wild-type; and v) PS-Peru (MRA190) used for *dhps*, A437G, K540E, A581G mutants. These materials were contributed by MR4/BEI Resources, NIAID, NIH, and were distributed by BEI Resources, NIAID, NIH. The use of these materials does not imply endorsement by the National Institutes of Health. The digested products were analyzed by 2% agarose (Eurogentec SA) gel electrophoresis in 1xTBE buffer (at 100 V for 1 h), and visualized by ultraviolet transilluminator after staining with ethidium bromide (Figure S1). Negative (water and human-negative DNA) controls were included in each experiment. Fragment sizes were estimated using a GeneRuler 100 bp DNA ladder (Thermo Fisher Scientific).

Reverse transcription quantitative PCR for detection of Pfs25 gametocyte-specific transcripts

One hundred μL of whole blood collected into 500 μL of RNAprotect stabilizer reagent (Qiagen, Hilden, Germany) were used to extract RNA using RNeasy Plus 96 Kit (Qiagen, Hilden, Germany) and eluted in 45 μl of the RNA Storage Solution (Ambion, Austin, TX, USA). Reverse transcription quantitative PCR (RT-qPCR) were performed as previously described [3]. Twenty μL reaction mixtures were run in a LightCycler® 480 Instrument (Roche Diagnostics, Basel, Switzerland) using the LightCycler MultiPlex RNA virus Master kit (Roche Diagnostics, Basel, Switzerland; 7083173001) with primers and HEX-BHQ1 labelled hydrolysis probe (250 mM, IDT, Coralville, IA, USA) published by Wampfler et al., (2013) [4]. All samples were tested in duplicate reactions and controls without reverse transcriptase (RT) enzyme were added to exclude false positives due to the presence of genomic DNA. The analysis was run using LightCycler480 software version 1.5.0. *P. falciparum* gametocyte densities were quantified using a 7-point standard curve ranging from 10^5 to 0.1 gametocytes/μL generated from cultured 3D7 *P. falciparum* stage V gametocytes as

described elsewhere [3]. The limit of detection (LOD) of this assay was 0.1 gametocytes/µL. Samples with Ct values higher than the last standard curve point, were considered to contain < 0.1 gametocytes/µL, *i.e.,* LOD.

FIGURES AND TABLES

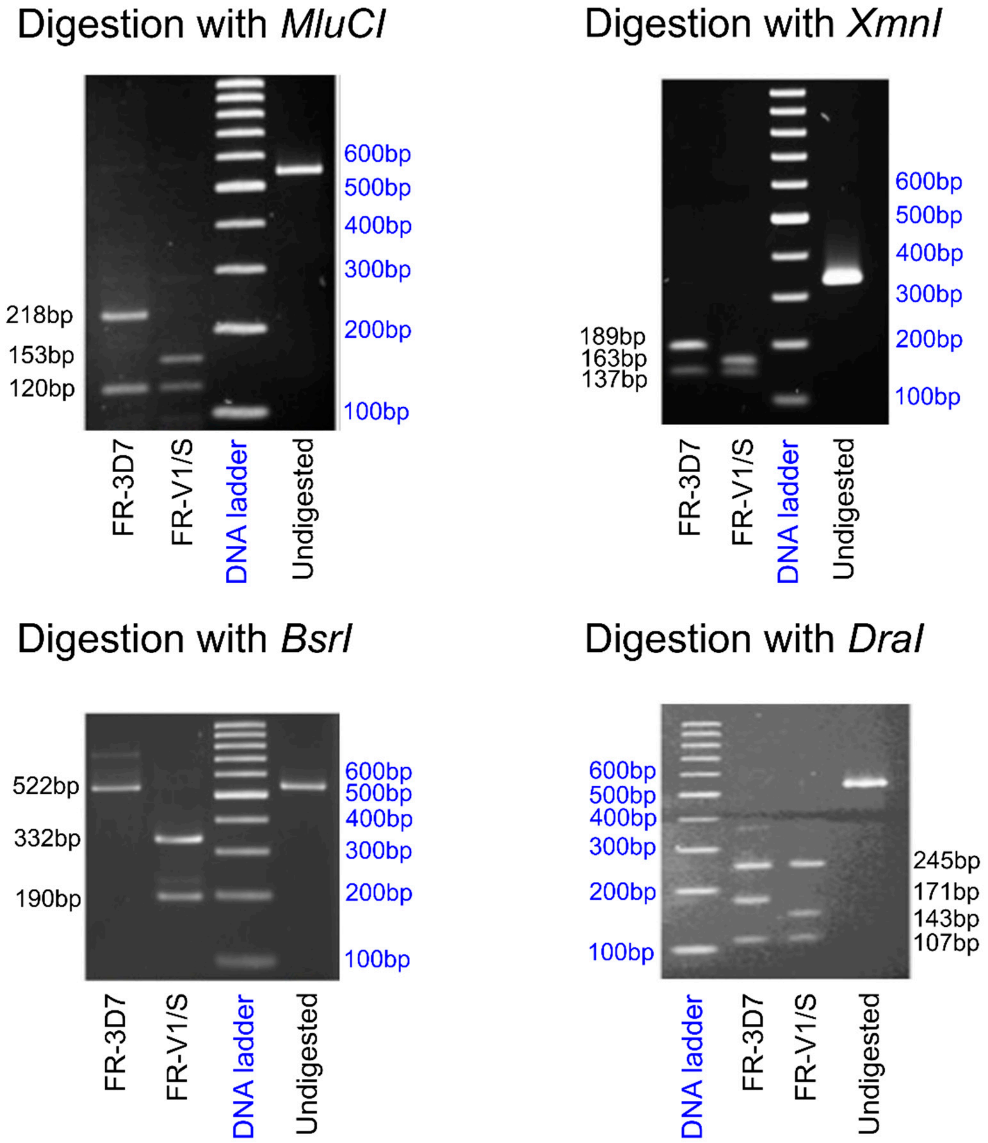

Figure S1. Visualization of *pfdhfr* and *pfdhps* genotyping results by PCR-restriction fragment length polymorphism (PCR-RFLP) using agarose gel electrophoresis. PCR fragments following digestion with the restriction enzymes MluCI XmnI, BsrI, and DraI are shown. The corresponding mutation codons targeted by each restriction enzyme are described in Table S1. The lane containing the molecular weight marker is indicated in blue as "DNA ladder", corresponding to the GeneRuler 100 bp DNA ladder (Thermo Fisher Scientific), which was used to estimate the digested fragment sizes (annotated in blue alongside the gel images). **Abbreviations and**

definitions: bp, base pairs; and FR-3D7 (MRA199) and FR-V1/S (MRA195) used as wild-type and mutant controls for *dhfr* N51I, C59R, S108N, I164L, respectively.

Table S1. Primer pairs and restriction enzymes used for *pfdhfr* and *pfdhps* polymorphism detection.

Primer name	Primer sequence	Size of amplicon (bp)	Targeted mutation	Restriction enzyme	Fragment length (bp) Wild Type	Fragment length (bp) Mutant
**AMP1/ AMP2**	5'-TTTATATTTTCTCCTTTTTA-3'<br>5'-CATTTTATTCGTTTTCT-3'	720				
M3-F/	5'- TTTATGATGGAACAAGTCTGCGACGTT- 3''<br>5' - AAATTCTTGATAAACAACGGAACCTttTA- 3'	522	N51I	*Mlu*CI	55, 65, 120, 153	55, 65, 120, 218
F-M4	5′-GAAATGTAATTCCCTAGATATGGAATATT-3′<br>5′-TTAATTTCCCAAGTAAAACTATTAGAGCTTC-3′	326	C59R	*Xmn*I	189, 137	26, 137, 163
M3-F/	5'- TTTATGATGGAACAAGTCTGCGACGTT- 3''<br>5' - AAATTCTTGATAAACAACGGAACCTttTA- 3'	522	S108N	*Bsr*I	522	190, 332
M3-F/	5'- TTTATGATGGAACAAGTCTGCGACGTT- 3''<br>5' - AAATTCTTGATAAACAACGGAACCTttTA- 3'	522	I164L	*Dra*I	107, 171, 245	107, 143, 245
**R2/R**	5'- AACCTAAACGTGCTGTTCAA- 3'<br>5' - AATTGTGTGATTTGTCCACAA-3'	710				
J-K	5'- TGCTAGTGTTATAGATATAGGatGAGcATC-3'<br>5’ – CTATAACGAGGTATTgCATTTAATgCAAGAA- 3'	438	S436F	*MspA*1I	438	32, 406
K-K/	5'- TGCTAGTGTTATAGATATAGGatGAGcATC-3'<br>5’ – CTATAACGAGGTATTgCATTTAATgCAAGAA- 3'	438	A437G	*Ava*II	438	34, 404
K-K/	5'- TGCTAGTGTTATAGATATAGGatGAGcATC-3'<br>5’ – CTATAACGAGGTATTgCATTTAATgCAAGAA- 3'	438	K540E	*Fok* I	33, 405	85, 320
L-L/ǂ	5' - ATAGGATACTATTTGATATTGGAccAGGATTcG-3'	161<br>161	A581G	*Bst*UI and *BslI**	105, 33, 23	138, 23

Table S2. Prevalence of *P. falciparum dhfr* and *dhps* haplotype combinations found at a low frequency.

Haplotype type	Mutation alleles	*n* (prevalence)
dhfr	51I/C59/108N/I164	5 (5%)
	51I/59R/S108/I1S1S1S164	1 (1%)
	51I/C59/S108/I164	1 (1%)
	N51/59R/108N/I164	3 (3%)
dhfr/dhps	108N+540E/581G	1 (1%)
	108N+163S	1 (1%)
	108N+163S	1 (1%)
	108N+WT	1 (1%)
	108N+437G/540E/581G	1 (1%)
	WT+437G/581G	1 (1%)
	59R/108N+WT	7 (7%)
	59R/108N+437G/540E	1 (1%)
	59R/108N+540E	5 (5%)
	59R/108N+581G	1 (1%)
	59R/108N+436F/437G	1 (1%)
	59R/108N+540E/163S	1 (1%)
	59R/108N/164L+540E	1 (1%)
	59R/108N/164L+437G/163S	1 (1%)
	59R+437G/540E	1 (1%)
	WT+WT	2 (2%)
	WT+437G/540E	1 (1%)
	WT+437G	1 (1%)
	WT+581G	1 (1%)
	WT+437G/540E/581G	1 (1%)
	WT+540E	1 (1%)
	WT+437G	1 (1%)
	59R/108N+437G/540E	1 (1%)
	59R/108N+WT	1 (1%)
	59R/108N+437G/540E/581G	1 (1%)

Abbreviations: *dhfr,* dihydrofolate reductase gene; *dhps*, dihydropteroate synthetase gene; WT, wild-type.

Table S3. Univariate analysis of risk factors associated with carriage of quintuple and sextuple mutant haplotypes (*n* = 100).

		Quintuple haplotype (*n* = 54) §				Sextuple haplotype (*n* = 7) ‡			
**Variable**	***n***	***n* [%]**	**OR**	**95% CI**	**p-*value***	***n* [%]**	**OR**	**95%CI**	**p-value**
Age (years)									
< 20	40	21 (52.5)	0.9	0.4-2.0	0.81	1 (2.5)	0.2	0.01-1.4	0.18
≥ 20	60	33 (61.1)		Ref.		6 (10.0)		Ref.	
Education									
None/primary	53	35 (66.0)	2.9	1.3-6.6	0.01	4 (7.6)	1.2	0.3-6.3	0.82
Secondary	47	19 (40.4)		Ref.		3 (6.4)		Ref.	
Place of residence									
Urban	57	26 (45.6)		Ref.		6 (10.5)		Ref.	
Rural	43	28 (65.1)	2.2	0.9-5.0	0.05	1 (2.3)	0.2	0.01-1.3	0.15
Gravidity									
Primigravidae (1)	45	22 (48.8)	0.7	0.3-1.5	0.35	2 (4.4)	0.5	0.1-2.3	0.38
Multigravidae (≥2)	55	31 (58.2)		Ref.		5 (9.1)		Ref.	
No. IPTp-SP doses received									
< 3 doses	46	27 (58.7)		Ref.		3 (6.5)		Ref.	
≥ 3 doses	54	27 (50.0)	1.4	0.6-3.2	0.39	4 (7.4)	0.9	0.2-4.1	0.86
*Parasite density, p/μL*Ψ									
<100 p/μL	67	37 (55.2)		Ref.		5 (7.5)		Ref.	
≥100 p/μL	33	17 (51.5)	0.9	0.9-1.9	0.73	2 (6.1)	0.8	0.1-4.4	0.77

Ψ Peripheral parasite density

§ Adjusted for age, education, residence location, gravidity, IPTp uptake and parasite density

‡ Adjusted for education, gravidity, IPTp uptake and parasite density

Statistical significance is indicated with $p < 0.05$.

Abbreviations: CI, confidence interval, IPTp-SP, intermittent preventive treatment for malaria in pregnancy with sulfadoxine-pyrimethamine, OR, odds ratio: Ref., Reference category.

Table S4. Effect of mutant haplotypes and other risk factors on adverse pregnancy outcomes in Chókwè district (*n* = 100) * (Univariate analysis).

Variable	*n*	BW < 2500g			Placental malaria ¥			Gestational age** (<37wk)		
		n [%]	OR [95%CI]	*p-value*	*n* [%]	OR [95%CI]	*p-value*	*n* [%]	OR [95%CI]	*p-value*
Mutant haplotype										
Sextuple‡	7	1 (14.3)	1.4 (0.1-15.3)	0.75	1 (14.3)	0.3 (0.03-2.7)	0.28	2 (28.6)	1.2(0.2-6.9)	0.87
Quintuple §	54	3 (5.6)	0.52 (0.1-2.4)	0.40	13 (24.1)	0.6 (0.2-1.3)	0.21	9 (16.7)	0.5 (0.2-1.6)	0.29
Others¶	39	4 (10.3)	Ref.		14 (35.9)	Ref.		10 (25.6)	Ref.	
Age (years)										
< 20	40	6 (15)	**5.1(0.9-26.7)**	**0.05**	13 (32.5)	1.4(0.6-3.5)	0.41	7 (17.5)	0.7(0.3-1.9)	0.48
≥ 20	60	2 (3.3)	Ref.		15 (25.0)	Ref.		14 (23.3)	Ref.	
Education										
None/primary	53	4 (3.7	0.8(0.2-4.8)	0.85	13 (24.5)	0.7(0.3-1.7)	0.41	10 (18.9)	0.7(0.3-1.)	0.56
Secondary	47	4 (8.5)	Ref.		15 (31.9)	Ref.		11 (23.4)	Ref.	
Place of residence										
Urban	57	5 (8.8	Ref.		18 (31.8)	Ref.		13 (24.1)	Ref.	
Rural	43	3 (6.9	0.78(0.2-3.4)	0.74	10 (23.3)	0.6(0.3-1.6)	0.36	8 (18.6)	0.7(0.3-2.1)	0.61
Gravidity										
Primigravidae (1)	45	8 (17.7)	N/A£	N/A	14 (31.1)	1.4(0.5-3.9)	0.54	6 (13.3)	0.4(0.1-1.2)	0.09
Multigravidae(≥ 2)	55	0 (0.0)	Ref.		14 (25.5)	Ref.		15 (27.3)	Ref.	
No. IPTp doses received										
< 3 doses	46	1 (2.2)	0.1(0.2-1.3)	0.08	13 (28.3)	1.0(0.4-2.5)	0.96	6 (13.0)	0.4(0.1-1.1)	0.08
≥ 3 doses	54	7 (13.0)	Ref.		15 (27.8)	Ref.		15 (27.8)	Ref.	

Statistical significance is indicated with $p < 0.05$. **¥** placental malaria as detected by histology, **£** analysis not done for birth weight due to observations in multigravidae. ** indicates gestational age at delivery, ***§*** (**IRN**-**GE**, **ǂ IRN-GEG, ¶** include WT and other mutant combinations**. Abbreviations:** BW, birth weight; CI, confidence interval; wk, weeks; IPTp-SP, intermittent preventive treatment for malaria in pregnancy with sulfadoxine-pyrimethamine; OR, odds ratio: Ref., Reference category.

Table S5. Parasite variability shown as Multiplicity of infection (MOI) in different mutation haplotypes

Variable	N	Median [IQR]	Type of infection		*p*-value
			Monoclonal (MOI = 1)	Polyclonal (MOI ≥ 2)	
**Mutations in participant**					
Wild-type	12	4.5 [2.5-5.5]	1	11	0.63
Any mutation	83	3 [2.0-4.0]	11	72	
**Mutant haplotype‡**					
Sextuple	5	3 [2.0-3.0]	0	5	0.13
Quintuple	54	3 [2.0-3.0]	10	44	
Other*	36	3.5 [2.5-5.0]	2	34	

*Composition of other mutations and combinations is presented in Table S2. **Abbreviations:** IQR, Interquartile range.

‡ two samples were excluded from the analysis due to inconclusive results for MOI assay.

Table S6. Frequency of gametocyte carriage and gametocyte densities by IPTp-SP uptake group.

	**IPTp-SP dose**					
	**Total**<br>**N = 100**	**None**<br>***n* = 12**	**One**<br>***n* = 11**	**Two**<br>***n* = 23**	**≥ 3**<br>***n* = 54**	***p*-value**
Gametocyte carrier	34	6	5	6	17	
% (n/N)	34.0	50.0	45.5	26.1	31.5	0.40
Median gametocyte density (*n*=15) *	1.76	0.66	0.15	1.86	1.87	0.39
Interquartile range [IQR]	[0.62-3.19]	[0.62-2.06]	[0.15-0.15]	[0.64-3.54]	[1.75-5.57]	

Gametocyte carriage was defined as presence of gametocytes detected by RTqPCR. Gametocyte densities were quantified by RT-qPCR, log10-transformed for regression analyses and raw values (per µL) are reported in this analysis. *Only 15 (44.1% [15/34]) samples could be quantified; 19 (55.9% [19/34]) had a gametocyte density of <0.1 gametocytes/µL. **Statistical tests** used to assess differences between IPTp-SP uptake groups: Chi-square (χ^2) test (group counts > 5) for gametocyte carriage, and Kruskal-Wallis test for gametocyte densities. **Abbreviations and definitions:** IPTp-SP, Intermittent preventive treatment in pregnancy with Sulfadoxine-Pyrimethamine; RT-qPCR, reverse transcription quantitative polymerase chain reaction

References

1. Duraisingh, M.T.; Curtis, J.; Warhurst, D.C. *Plasmodium falciparum:* detection of polymorphisms in the dihydrofolate reductase and dihydropteroate synthetase genes by PCR and restriction digestion. *Exp Parasitol* **1998**, *89*, https://doi.org/10.1006/expr.1998.4274.
2. Plowe, C.V.; Cortese, J.F.; Djimde, A.; Nwanyanwu, O.C.; Watkins, W.M.; Winstanley, P.A.; Estrada-Franco, J.G.; Mollinedo, R.E.; Avila, J.C.; Cespedes, J.L.; et al. Mutations in *Plasmodium falciparum* dihydrofolate reductase and dihydropteroate synthase and epidemiologic patterns of pyrimethamine-sulfadoxine use and resistance. *J. Infect. Dis.* **1997**, *176*, 1590–1596, https://doi.org/10.1086/514159.
3. Rovira-Vallbona, E.; Contreras-Mancilla, J.J.; Ramirez, R.; Guzmán-Guzmán, M.; Carrasco-Escobar, G.; Llanos-Cuentas, A.; Vinetz, J.M.; Gamboa, D.; Rosanas-Urgell, A. Predominance of asymptomatic and sub-microscopic infections characterizes the *Plasmodium* gametocyte reservoir in the Peruvian Amazon. *PLoS Negl. Trop. Dis.* **2017**, *11*, e0005674, https://doi.org/10.1371/journal.pntd.0005674.
4. Wampfler, R.; Mwingira, F.; Javati, S.; Robinson, L.; Betuela, I.; Siba, P.; Beck, H.P.; Mueller, I.; Felger, I. Strategies for detection of *Plasmodium* species gametocytes. *PLoS One* **2013**, *8*, e76316, https://doi.org/10.1371/journal.pone.0076316.
